# Novel multi-strain probiotics reduces *Pasteurella multocida* induced fowl cholera mortality in broilers

**DOI:** 10.1038/s41598-021-88299-0

**Published:** 2021-04-26

**Authors:** Rine Christopher Reuben, Shovon Lal Sarkar, Habiba Ibnat, Md. Ali Ahasan Setu, Pravas Chandra Roy, Iqbal Kabir Jahid

**Affiliations:** 1 Department of Microbiology, Faculty of Biological Science and Technology, Jashore University of Science and Technology, Jashore, 7408 Bangladesh; 2 German Centre for Integrative Biodiversity Research (iDiv), Halle-Jena-Leipzig, Germany

**Keywords:** Microbiology, Applied microbiology

## Abstract

*Pasteurella multocida* causes fowl cholera, a highly contagious poultry disease of global concern, causing significant ecological and economic challenges to the poultry industry each year. This study evaluated the effects of novel multi-strain probiotics consisting of *Lactobacillus plantarum*, *L. fermentum*, *Pediococcus acidilactici*, *Enterococcus faecium* and *Saccharomyces cerevisiae* on growth performance, intestinal microbiota, haemato-biochemical parameters and anti-inflammatory properties on broilers experimentally challenged with *P. multocida*. A total of 120 birds were fed with a basal diet supplemented with probiotics (10^8^ CFU/kg) and then orally challenged with 10^8^ CFU/mL of *P. multocida*. Probiotics supplementation significantly (P < 0.05) improved growth performance and feed efficiency as well as reducing (P < 0.05) the population of intestinal *P. multocida*, enterobacteria, and mortality. Haemato-biochemical parameters including total cholesterol, white blood cells (WBC), proteins, glucose, packed cell volume (PCV) and lymphocytes improved (P < 0.05) among probiotic fed birds when compared with the controls. Transcriptional profiles of anti-inflammatory genes including hypoxia inducible factor 1 alpha (HIF1A), tumor necrosis factor- (TNF) stimulated gene-6 (TSG-6) and prostaglandin E receptor 2 (PTGER2) in the intestinal mucosa were upregulated (P < 0.05) in probiotics fed birds. The dietary inclusion of the novel multi-strain probiotics improves growth performance, feed efficiency and intestinal health while attenuating inflammatory reaction, clinical signs and mortality associated with *P. multocida* infection in broilers.

## Introduction

*Pasteurella multocida* is a Gram-negative coccobacillus and readily transmitted bacterium which causes fowl or avian cholera, an acute and fatal septicemic infection affecting wide range of both wild and domestic bird species^1,2^. In poultry, it causes significant economic loss on back-yard and large-scale commercial poultry production globally^3,4^. Although not common, human cases of *P. multocida* infections are often associated with immunosuppressed individuals, older adults or rarely due to occupational exposure^5,6^. The route of infection and pathogenesis of *P. multocida* in poultry have not been clearly elucidated, however, accumulated evidence suggests the respiratory tract as the major entry point^2^. Furthermore, *P. multocida* is known to be normal microflora of the upper respiratory tract (URT) of most healthy animals including poultry, and it can inhabit the oropharynx of healthy hosts for elongated periods without causing disease^2^. However, virulent strains of *P. multocida* are able to colonize the mucosa of the upper respiratory tract and, subsequently, infect the air sacs and lungs of birds. Through an unknown mechanism, but possibly related to the migration in macrophages of the upper respiratory tract, the bacteria can access the blood circulation from the mucosa and multiply in different tissues, especially in the liver and spleen^2,4^.

In the poultry industry, antibiotics have been used over the years in the treatment and control of poultry infections and in some countries also as growth promoters. The control of *P. multocida* infections in poultry using antibiotics has been somewhat successful, nevertheless, there is often relapse after antibiotic withdrawal. More so, 80.5% of *P. multocida* infections have shown high degree of resistance to broad range of commonly used antibiotics^7,8^. Strains of *P. multocida* from ducks, chickens, geese, turkeys and quails have reportedly shown resistance to doxycycline-HCl, enrofloxacin, chloramphenicol, norfloxacin and lincomycin^4,8,9^.

With the phasing out of antibiotics in animal production due to immense public health concerns including the presence of drug residue in animal products, emergence and spread of resistance, dysbiosis of gut microflora and hypersensitivity among others, there is need for the application of naturally safe alternatives which will both improve animal growth performance as well as control infectious diseases. Probiotics, which are now widely accepted as alternatives to antibiotics, are viable microorganisms which confer wide range of nutritional and health benefits in animals when administered in sufficient amount. Probiotics ameliorate enteric infections through competitive exclusion of pathogens, and chronic inflammatory and allergic diseases, as well as immunomodulation and immune-stimulation, increased digestibility and nutrients assimilation in their host^10–13^.

The regular inclusion of probiotics in poultry diets may both minimize the risk of infections with pathogens such as *P. multocida*, *E. coli*, *Campylobacter* spp., *Listeria monocytogenes* and *Salmonella* as well as improving growth performance in birds^14^. This would significantly decrease the risks associated with poultry and poultry product contamination with animal pathogens of public health concern, hence reducing human spread as well as safeguarding the environment.

Recently, several successful reports have emerged on the treatment of poultry infections and multidrug resistant bacterial pathogens using probiotic strains^13–18^. Up to date, apart from few field studies on the effectiveness of antibiotic alternatives such as bacteriophages, vaccines, β-glucan, and certain proteins against *P. multocida* infections^19–23^, no study has evaluated the effectiveness of probiotics in ameliorating infections caused by *P. multocida* in poultry despite its devastating effects in the poultry industry. Being known as an ‘inscrutable’ pathogen, some strains of *P. multocida* have the potential of colonizing the nasopharynx, respiratory and the gastrointestinal tracts of animals including poultry thereby inducing severe disease. The use of probiotics in mitigating infection caused by *P. multocida*, which is rarely regarded as a gut-associated pathogen, would further aid in reducing the intestinal colonization and/or severity infection by other gut-associated and opportunistic pathogens.

In our previous studies, strains of LAB (lactic acid bacteria) were isolated, characterized and evaluated for antagonistic activity against poultry pathogens and in vitro probiotic properties^17,18^. We hypothesized that dietary multi-strains probiotic supplementation would improve the growth performance, modulate intestinal microflora and haemato-biochemical parameters as well as ameliorate *P. multocida* infection in broiler chickens. This study aimed to investigate the effect of dietary supplementation with novel multi-strain probiotic (consisting of *Lactobacillus plantarum, L. fermentum, Pediococcus acidilactici, Enterococcus faecium and Saccharomyces cerevisiae*) on the growth performance, intestinal microflora, haemato-biochemical parameters and anti-inflammatory properties on *P. multocida* infection in broilers*.*

## Results

### Growth performance

From the beginning of this experimental trial, i.e., days 1 to 14, no statistically significant (P > 0.05) differences were recorded in the body weight (BW), body weight gain (BWG), feed intake (FI) and feed conversion ratio (FCR) among all the treatments. The FCR differs significant (P < 0.05) between the probiotic supplemented groups (Pro− and Pro+) when compared with the with the NC− while other groups showed non-significantly higher values (Table 1). During the post—*P. multocida* challenge period, i.e., days 14 to 28, probiotics supplementation significantly (P < 0.05) affected growth performance and feed utilization (Table 1). There was severe depression in the growth, FI and FCR in the NC+ and PC+ treatments in the first- and second-week post infection compared with other treatments. In general, probiotics supplemented groups, with or without *P. multocida* challenged showed significantly (P < 0.05) higher BW and BWG with feed efficiency than other treatments at the end of the experiment. Birds in the challenged positive control (PC+) and the challenged negative control (NC+) treatments had the worse BW, BWG and FCR compared to others from day 21 onwards (Table 1).

**Table 1:** Effects of probiotics supplementation on growth performance of *Pasteurella multocida* challenged broiler chickens.

Growth performance	Treatment							
	NC−	PC−	Pro−	Pro+	PC+	NC+	SEM	*P* value
BW 1 (g)	47.00^a^	46.86^a^	46.55^a^	46.95^a^	47.05^a^	46.75^a^	0.145	0.071
BW 7 (g)	143.85^a^	139.05^a^	145.7^a^	141.1^a^	142.7^a^	142.65^a^	0.931	0.087
BWG (g)	96.85^a^	92.45^a^	99.15^a^	94.15^a^	95.25^a^	96.1^a^	0.941	0.999
FI (g)	231^a^	239.2^a^	234.45^a^	238.15^a^	253.7^a^	230.1^a^	3.520	0.105
FCR	2.385^a^	2.587^a^	2.365^a^	2.529^a^	2.664^a^	2.394^a^	0.051	0.083
BW 14 (g)	324.2^b^	329.95^b^	356.45^ab^	352.1^ab^	338.7^b^	320.7^b^	6.024	0.041
BWG (g)	277.2^a^	283.67^a^	309.9^ab^	305.15^ab^	291.25^a^	274.15^a^	6.005	0.050
FI (g)	435.5^ab^	405^ac^	410.5^ab^	401.4^ad^	400.6^ae^	397.8^aef^	5.694	0.012
FCR	1.571^a^	1.428^ab^	1.325^b^	1.315^b^	1.375^b^	1.451^ab^	0.039	0.031
BW 21 (g)	621.65^c^	631.69^c^	687.2^a^	651.26^b^	426.36^d^	414.89^d^	48.811	0.015
BWG (g)	574.65^c^	585.11^c^	640.65^a^	604.21^b^	379.81^d^	368.11^d^	48.792	0.032
FI (g)	957.00^a^	903.58^d^	804.07^c^	701.11^d^	641.73^e^	662.44^f^	53.643	0.029
FCR	1.665^b^	1.544^c^	1.255^d^	1.160^d^	1.690^b^	1.799^a^	0.105	0.044
BW 28 (g)	989.22^c^	942.18^d^	1201.72^a^	1195.71^b^	612.38^e^	582.57^f^	110.928	0.009
BWG (g)	942.22^c^	895.58^d^	1155.17^a^	1148.76^b^	564.93^e^	536.02^f^	110.969	0.010
FI (g)	1139.55^a^	993.55^b^	1096.53^b^	999.05^b^	865.17^c^	699.46^d^	65.883	0.011
FCR	1.209^c^	1.109^d^	0.949^e^	0.870^f^	1.531^a^	1.305^b^	0.099	0.025

Values are means of two replicates and standard errors of means.

Within each variable, values with the same superscript letter are not significantly different according to Duncan’s multiple range test (P > 0.05).

NC−: unchallenged negative control; PC−: unchallenged positive control; Pro−: unchallenged probiotics control; Pro+: challenged probiotic control; PC+: challenged positive control; NC+: challenged negative control, BW−: body weight; BWG−: body weight gain; F−: feed intake; FCR: feed conversion ration.

### Clinical signs and mortality

**Figure 1:**
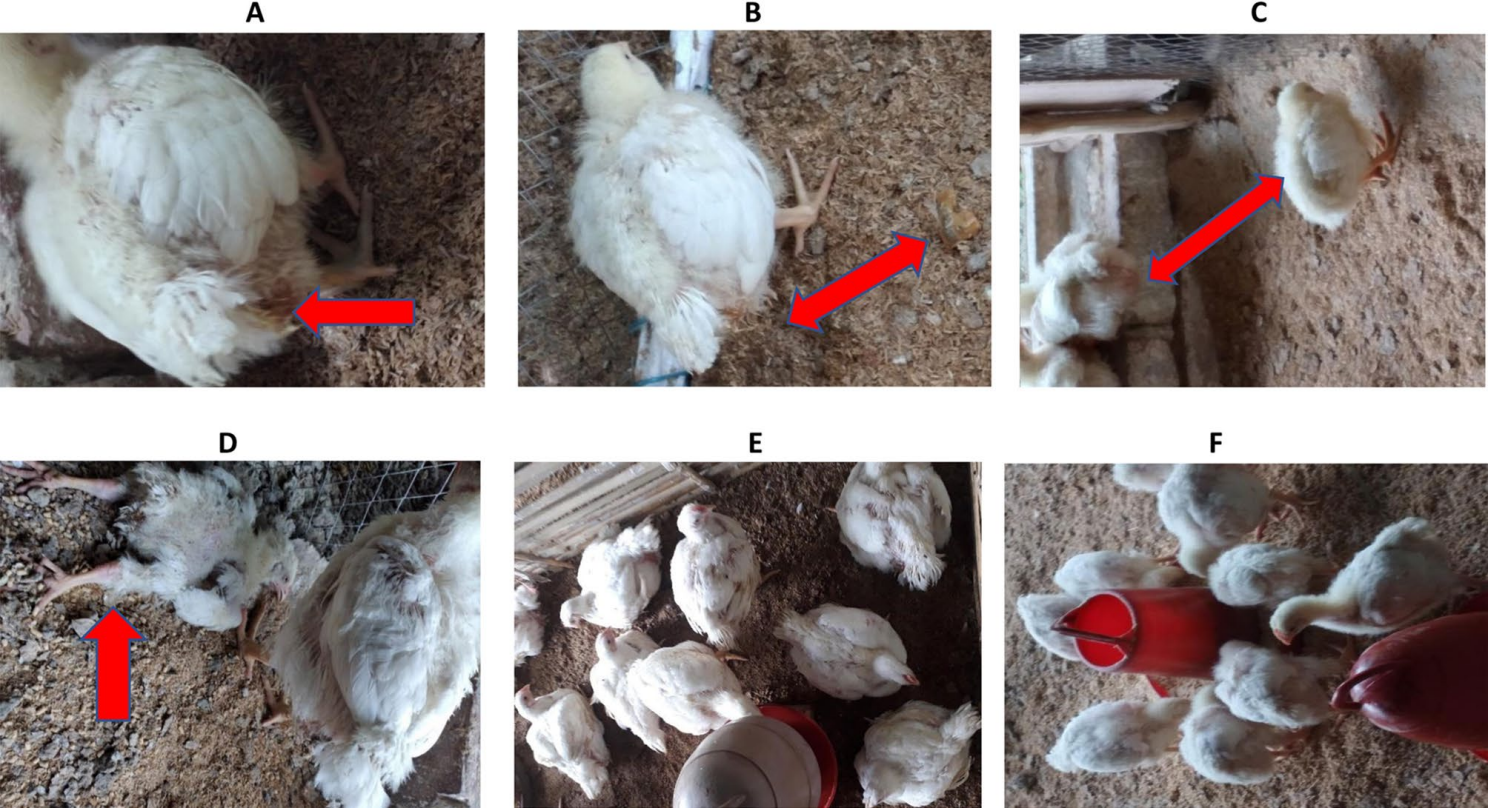
Symptoms in *Pasteurella multocida* challenged groups supplemented with antibiotic (PC+) and control (NC+); (**A**) Yellowish-grey diarrhoea appeared on the cloaca, (**B**) Yellowish-grey diarrhoea appeared on the cloaca and littered, (**C**) Depression and isolation, (**D**) Moribund, lameness and ruffled feathers, (**E**) Healthy and active probiotics supplemented broilers challenged with *Pasteurella multocida* (Pro+), (**F**) Healthy and active probiotics supplemented broilers challenged with *Pasteurella multocida* (Pro+).

In a pilot study to determine the infectivity, broilers were challenged with *P. multocida* and the clinical signs and death due to *P. multocida* were recorded (data not shown). Severe pasteurellosis due to the experimental inoculation of birds with *P. multocida* was initially induced in the NC+ group and later in the PC+ group about 12 and 24 h post-challenged (Fig. 1). Clinical signs manifested in challenged birds included diarrhoea, depression, severe weakness, nasal discharge, isolation, reduction in feed and water consumption, ruffled feathers, immobility and lameness which are the characteristics of *P. multocida* infection in poultry (Fig. 1). Although the most severe clinical manifestations recorded in this study were observed between 24 to 94 h post-infection, mild to moderate clinical signs persist in the challenged groups (PC+ and NC+) until the end of the experiment while no obvious clinical signs were observed in challenged probiotic (Prob+) group (Fig. 1). At the end of the trial, mortality rates attributed to *P. multocida* infection were 5.00, 60.00 and 65.00% for Pro+, PC+ and NC+ treatments respectively (Table 2). Mortality was significantly higher (P < 0.05) in birds in the PC+ and NC+ groups when compared with the Pro+ group. Similarly, the highest mortality recorded due to *P. multocida* occurred during the first 72 h post-infection, with 10 and 8 mortalities in PC+ and NC+ groups while the lone mortality recorded in Prob+ throughout the entire study occurred on 72 h post infection (Table 2).

**Table 2:** Mortality rate of broiler chickens supplemented with probiotics and challenged with *Pasteurella multocida*.

Days	NC−	PC−	Pro−	Pro+	PC+	NC+
1	0	0	0	0	0	0
2	0	0	0	0	2	3
3	0	0	0	0	4	2
4	0	0	0	1	4	3
5	0	0	0	0	0	3
6	0	0	0	0	1	1
7	0	0	0	0	0	0
8	0	0	0	0	0	0
9	0	0	0	0	0	0
10	0	0	0	0	0	0
11	0	0	0	0	1	0
12	0	0	0	0	0	0
13	0	0	0	0	0	1
14	0	0	0	0	0	0
Total	0	0	0	1	12	13
% mortality	0^c^	0^c^	0^c^	5^b^	60^a^	65^a^

Within each variable, values with the same superscript letter are not significantly different according to Duncan’s multiple range test (P > 0.05). NC−: unchallenged negative control; PC−: unchallenged positive control; Prob−: unchallenged probiotics control; Pro+: challenged probiotic control; PC+: challenged positive control; NC+: challenged negative control.

### Carcass and visceral organs weight

On day 21 of the experiment, the relative weight of the Bursa in the probiotics supplemented birds (Pro− and Pro+) were significantly (P < 0.05) higher when compares to birds in other treatments, with the NC+ having the least weight of 0.09 g as recorded. Similarly, the relative weight of ileum in Prob+ group differed significantly (P < 0.05) when compared with other *P. multocida* challenged groups (PC+ and NC+), and non challenged groups (PC− and NC−) (Table 3). The relative weights of the wing and dressing carcass recorded from birds in Pro− and Pro+ groups were significantly (P < 0.05) higher than NC+. Furthermore, whereas the relative weight of the liver from birds in Pro− group was significantly higher than birds from all the groups except Pro+ (which was numerically higher) on day 28, the relative weight of the spleen in the NC+ and PC− groups were significantly lower when compared with other groups. Also, although the dressing carcass of the PC− group was significantly (P < 0.05) higher than all other groups (except Pro+), the dressing carcass of birds in the Pro+ group similarly had significantly (P < 0.05) higher relative weight when compared with other challenged groups (PC+ and NC+) (Table 3). The relatively weights of carcass and visceral organs from the *P. multocida* challenged groups generally had the least values especially from birds in the NC+ group.

**Table 3:** Relative weights (% BW) of organs from broilers supplemented with probiotics and challenged with *Pasteurella multocida*.

Organ	Treatment							*P* value
	NC−	PC−	Pro −	Pro+	PC+	NC+	SEM	
**Day 21**								
Heart	0.74^a^	0.54^a^	0.74^a^	0.72^a^	0.69^a^	0.53^a^	0.040	0.298
Liver	2.083^a^	2.36^a^	2.99^a^	3.29^a^	2.77^a^	2.53^a^	0.179	0.107
Spleen	0.11^a^	0.17^a^	0.25^a^	0.19^a^	0.13^a^	0.09^a^	0.013	0.929
Bursa	0.12^c^	0.13^c^	0.30^ab^	0.35^a^	0.18^bc^	0.09^c^	0.043	0.021
Gizzard	3.46^a^	3.11^a^	3.54^a^	3.82^a^	3.50^a^	3.61^a^	0.094	0.183
Ileum	2.09^b^	2.24^b^	2.57^b^	3.53^a^	2.75^b^	2.73^b^	0.207	0.021
Caecum	0.57^a^	0.46^a^	0.58^a^	0.71^a^	0.63^a^	0.38^a^	0.048	0.092
Thigh	4.77^a^	4.02^a^	4.07^a^	4.28^a^	4.42^a^	4.25^a^	0.111	0.202
Drumstick	4.34^a^	3.63^a^	3.86^a^	3.58^a^	3.34^a^	3.41^a^	0.149	0.075
Breast	19.57^ab^	20.15^ab^	21.98^a^	19.60^ab^	17.30^bc^	13.75^c^	1.060	0.041
Wing	2.13^ab^	1.81^ab^	2.67^a^	2.02^ab^	1.90^ab^	1.56^b^	0.096	0.012
Dressing	55.07^ab^	54.62^ab^	61.47^a^	57.53^ab^	45.22^bc^	41.37^c^	1.720	0.023
**Day 28**								
Heart	0.55^a^	0.43^a^	0.61^a^	0.63^a^	0.63^a^	0.63^a^	0.032	0.081
Liver	2.61^b^	2.54^b^	4.45^a^	3.59^ab^	2.60^b^	2.63^b^	0.108	0.016
Spleen	0.13^ab^	0.02^c^	0.17^a^	0.18^a^	0.15^a^	0.07^bc^	0.024	0.028
Bursa	0.46^ab^	0.45^ab^	0.94^a^	0.75^ab^	0.33^b^	0.40^ab^	0.036	0.011
Gizzard	2.92^a^	2.69^a^	2.43^a^	2.83^a^	3.61^a^	3.33^a^	0.175	0.083
Ileum	3.10^a^	2.29^bc^	3.44^a^	2.89^ab^	2.71^ab^	1.88^c^	0.195	0.001
Caecum	0.65^ab^	0.46^b^	0.77^ab^	1.07^a^	1.02^ab^	0.52^ab^	0.103	0.031
Thigh	4.92^a^	4.56^a^	4.90^a^	4.28^ab^	3.18^c^	3.48^bc^	0.279	0.012
Drumstick	4.35^ab^	4.16^ab^	4.93^a^	4.24^ab^	3.38^b^	3.11^b^	0.138	0.031
Breast	19.83^ab^	18.05^ab^	21.25^a^	18.59^ab^	16.72^ab^	15.03^b^	0.900	0.007
Wing	2.62^ab^	2.56^ab^	2.16^a^	2.57^ab^	2.75^a^	2.46^ab^	0.082	0.039
Dressing	56.27^bc^	56.58^bc^	66.30^a^	64.64^ab^	49.00^c^	47.65^c^	2.593	0.011

Values are means of two replicates and standard errors of means.

Within each variable, values with the same superscript letter are not significantly different according to Duncan’s multiple range test (P > 0.05).

NC−: unchallenged negative control; PC−: unchallenged positive control; Pro−: unchallenged probiotics control; Pro+: challenged probiotic control; PC+: challenged positive control; NC+: challenged negative control.

### Enumeration of intestinal digesta intestinal bacteria, yeast and *P. multocida*

There was no significant (P > 0.05) differences in the numbers of total aerobes from the ileal and caecal contents on day 21 of the trial among all the treatments. However, the LAB counts in the gizzard contents of probiotics supplemented birds (groups Pro− and Pro+) was significant (P < 0.05) higher on day 28 when compared with other groups (Table 4). Also, the LAB counts in the ileum and caecum of birds from PC+ and NC+ were lower (P < 0.05) than probiotics supplemented birds (groups Pro− and Pro+). The numbers of enterobacteria recorded from the gizzard, ileum and caecum on day 21 of the trial were significantly (P < 0.05) higher in *P. multocida* challenged groups, PC+ and NC+ , when compared with other treatments, and this trend persist till the end of the trial. The numbers of yeast cells tended to be higher in the ileum and caecum of birds supplemented probiotics than other groups. However, the least yeast counts were recorded from the gizzards of all the birds across the treatments with birds in PC+ and NC+ groups having a significantly (P < 0.05) lowered counts on day 21.

**Table 4:** Microbial counts (Log_10_ cfu/g) in the digesta of chickens supplemented with probiotics and challenged with *Pasteurella multocida*.

Content	Treatment							P-value
	NC−	PC−	Pro−	Pro+	PC+	NC+	SEM	
**Day 21**								
Gizzard								
Total aerobes	8.45^ab^	6.87^c^	8.53^ab^	9.39^a^	7.91^bc^	8.47^ab^	0.345	0.008
Enterobacteria	4.80^ab^	4.74^ab^	3.54^b^	3.54^b^	5.19^a^	5.30^a^	0.329	0.017
LAB	7.55^a^	7.37^a^	8.11^a^	8.48^a^	7.39^a^	7.91^a^	0.181	0.205
Yeast	6.48^a^	6.73^a^	6.65^a^	6.66^a^	5.70^b^	5.60^b^	0.210	0.018
*P. multocida*	0.00^b^	0.00^b^	0.00^b^	0.50^b^	5.54^a^	5.20^a^	1.109	0.001
Ileum								
Total aerobes	8.78^a^	7.77^a^	9.06^a^	8.95^a^	8.35^a^	8.46^a^	0.194	0.402
Enterobacteria	5.64^a^	5.73^a^	3.99^b^	3.48^b^	5.40^a^	5.86^a^	0.379	0.020
LAB	8.25^ab^	7.75^b^	9.35^a^	9.11^ab^	7.87^ab^	8.07^ab^	0.273	0.015
Yeast	7.66^a^	8.22^a^	8.85^a^	8.41^a^	7.87^a^	7.90^a^	0.177	0.082
*P. multocida*	0.00^c^	0.00^c^	0.00^c^	2.54^b^	5.85^a^	5.87^a^	1.172	0.000
Caecum								
Total aerobes	9.55^a^	9.00^a^	9.67^a^	9.08^a^	8.69^a^	9.09^a^	0.149	0.087
Enterobacteria	5.84^a^	6.29^a^	3.06^b^	3.33^b^	6.22^a^	6.51^a^	0.438	0.017
LAB	8.54^b^	8.99^ab^	9.69^a^	9.28^ab^	8.65^b^	8.59^b^	0.187	0.014
Yeast	8.54^c^	8.99^bc^	9.68^a^	9.26^ab^	7.15^d^	7.61^d^	0.403	0.001
*P. multocida*	0.00^c^	0.00^c^	0.00^c^	3.65^b^	6.86^a^	6.40^a^	1.338	0.010
**Day 28**								
Gizzard								
Total aerobes	8.34^ab^	7.37^b^	9.45^a^	9.44^a^	8.22^ab^	9.20^a^	0.341	0.041
Enterobacteria	4.80^b^	4.89^b^	3.03^c^	2.98^c^	5.45^ab^	5.92^a^	0.415	0.025
LAB	8.45^bc^	7.83^c^	8.93^b^	9.96^a^	7.77^c^	8.36^bc^	0.331	0.012
Yeast	7.26^a^	7.13^a^	7.29^a^	7.00^a^	6.00^a^	7.20^a^	0.201	0.218
*P. multocida*	0.00^c^	0.00^c^	0.00^c^	1.50^b^	4.65^a^	4.74^a^	0.941	0.013
Ileum								
Total aerobes	8.26^ab^	7.74^b^	9.31^a^	9.55^a^	8.23^ab^	8.75^ab^	0.283	0.041
Enterobacteria	6.22^b^	6.34^ab^	2.41^c^	3.06^c^	6.60^ab^	7.04^a^	0.448	0.000
LAB	7.75^c^	8.25^abc^	9.35^a^	9.14^ab^	8.18^bc^	8.20^bc^	0.253	0.002
Yeast	8.22^b^	7.69^b^	9.47^a^	8.23^b^	7.15^b^	7.69^b^	0.324	0.040
*P. multocida*	0.00^c^	0.00^c^	0.00^C^	2.65^b^	6.72^a^	7.01^a^	1.126	0.001
Caecum								
Total aerobes	9.35^b^	9.00^c^	9.89^a^	9.67^a^	9.10^c^	8.62^d^	0.193	0.042
Enterobacteria	6.81^ab^	6.17^b^	3.13^c^	3.52^c^	7.13^ab^	7.81^a^	0.514	0.007
LAB	8.54^b^	8.48^b^	9.84^a^	9.69^a^	8.92^b^	8.47^b^	0.254	0.039
Yeast	7.75^b^	8.33^b^	9.93^a^	8.70^ab^	7.61^a^	7.75^a^	0.359	0.043
*P. multocida*	0.00^c^	0.00^c^	0.00^c^	3.28^b^	6.53^a^	6.62^a^	1.332	0.006

Values are means of two replicates and standard errors of means. Within each variable, values with the same superscript letter are not significantly different according to Duncan’s multiple range test (P > 0.05).

NC−: unchallenged negative control; PC−: unchallenged positive control; Pro−: unchallenged probiotics control; Pro+: challenged probiotic control; PC+: challenged positive control; NC+: challenged negative control.

Throughout this experimental trial, birds in the probiotic control, Pro+ treatment had significantly (P < 0.05) lowered counts of *P. multocida* in their gizzards, ilea and caeca when compared with those of birds in the other challenged treatments, PC+ and NC+ respectively (Table 4). Whereas the gizzards of birds in the probiotic control treatment, Prob+ had the least counts of *P. multocida,* 0.50 log_10_CFU/g on day 21, significantly higher counts of *P. multocida* ranging between 6.62 and 7.01 log_10_CFU/g were recorded from the ilea and caeca of birds in the PC+ and NC+ treatments on day 28 of the trial. The numbers of *P. multocida* in the ileal and caecal contents were significantly (P < 0.05) reduced in Pro+ group when compared with other challenged groups, PC+ and NC+ on days 21 and 28 (i.e., 7 and 14 days post-challenged) respectively. Birds from all the treatments were confirmed to be culture-negative for *Pasteurella* before inoculation with *P. multocida* on day 14 of the experiment. Also, the non-*P. multocida* challenged treatments (i.e., NC−, PC− and Pro−) were *Pasteurella* negative throughout the experimental period.

### Intestinal digesta pH

Generally, this study recorded an increasing pH concentration from acidity to neutrality with the gizzard digesta of birds having the lowest pH levels followed by the ileal and then the caecal contents respectively (Table 5). The pH of the ileal contents from birds in PC+ groups were significantly (P < 0.05) lowered when compared with other challenged groups, PC+ and NC+ on days 21 and 28 respectively.

**Table 5:** Effects of supplementation with probiotics and challenged with *Pasteurella multocida* on pH of GIT of broilers.

pH	NC −	PC −	Pro −	Pro+	PC+	NC+	SEM	P-value
**Day 21**								
Gizzard	3.53^ab^	3.84^ab^	3.18^b^	3.38^b^	3.75^ab^	4.1^b^	0.137	0.057
Ileum	5.62^a^	5.83^a^	4.71^bc^	3.93^c^	5.7^a^	5.61^ba^	0.298	0.021
Caecum	6.47^a^	6.14^a^	6.17^a^	5.89^a^	6.04^a^	6.27^a^	0.139	0.287
**Day28**								
Gizzard	3.63^a^	3.80^a^	3.01^a^	2.95^a^	3.76^a^	3.65^a^	0.155	0.093
Ileum	5.73^ab^	5.97^a^	5.36^ab^	5.09^b^	5.71^ab^	6.05^a^	0.119	0.026
Caecum	7.15^a^	6.72^a^	6.43^a^	6.31^a^	6.64^a^	6.65^a^	0.115	0.105

Values are means of two replicates and standard errors of means.

Within each variable, values with the same superscript letter are not significantly different according to Duncan’s multiple range test (P > 0.05).

NC−: unchallenged negative control; PC−: unchallenged positive control; Pro−: unchallenged probiotics control; Pro+: challenged probiotic control; PC: challenged positive control; NC+: challenged negative control.

### Haemato-biochemical parameters

The total cholesterol concentration in birds from the probiotics supplemented groups, Pro− and Pro+ were significantly (P < 0.05) lower than *P. multocida* challenged groups, PC+ and NC+ on days 21 and 28 of the experiment. Nevertheless, there was no significant (P > 0.05) difference in the HDL cholesterol and LDL cholesterol concentrations across the treatment all through the experiment. On day 28, while triglyceride levels were reduced (P > 0.05) in probiotics supplemented groups but higher (P < 0.05) in NC−, glucose levels were significantly(P < 0.05) reduced in the same groups. Similarly, protein levels were significantly (P < 0.05) higher in probiotics supplemented groups when compared with other groups on day 28 (Table 6).

**Table 6:** Effects of probiotics supplementation on biochemical parameters of *Pasteurella multocida* challenged broiler chickens.

Parameter	Treatment							*P* value
	NC−	PC−	Pro−	Pro+	PC+	NC+	SEM	
**Day 21**								
Total cholesterol (mg/dL)	92^ab^	94.04^ab^	74.00^b^	81.5^b^	111^a^	108^a^	4.333	0.024
HDL cholesterol (mg/dL)	76^a^	74^a^	72.5^a^	82.5^a^	91^a^	93^a^	4.197	0.095
LDL cholesterol (mg/dL)	26^a^	20.5^a^	24.5^a^	22^a^	24^a^	25.5^a^	0.864	0.217
Triglyceride (mg/dL)	56.5^a^	60^a^	54.5^a^	52^a^	70^a^	60^a^	3.829	0.089
Total cholesterol-HDL ratio	1.18^a^	1.25^a^	1.23^a^	1.23^a^	1.22^a^	1.16^a^	0.014	0.101
Total protein (g/dl)	2.53^bc^	2.8^abc^	3.26^a^	2.95^ab^	2.52^bc^	2.25^c^	0.114	0.021
Glucose (mmol/L)	12.34^a^	13.65^a^	11.12^a^	11.38^a^	14.18^a^	13.64^a^	0.386	0.082
**Day 28**								
Total cholesterol (mg/dL)	100.5^a^	110.5^a^	76.5^b^	79^b^	105.5^a^	107^a^	5.581	0.031
HDL cholesterol (mg/dL)	66^a^	91^a^	68^a^	73.5^a^	73^a^	86^a^	4.098	0.097
LDL cholesterol (mg/dL)	28.6^a^	30.5^a^	20.7^a^	27^a^	37.5^a^	31.5^a^	2.260	0.310
Triglyceride (mg/dL)	113.5^a^	111.5^ab^	71.5^b^	91^ab^	103^ab^	105^ab^	9.681	0.021
Total cholesterol-HDL ratio	1.27^a^	1.21^a^	1.09^a^	1.20^a^	1.27^a^	1.26^a^	0.027	0.472
Total protein (g/dL)	2.81^bc^	2.89^bc^	3.64^a^	3.51^ab^	2.25^c^	2.38^c^	0.232	0.009
Glucose (mmol/L)	12.21^a^	15.05^a^	9.04^b^	8.17^b^	13.87^a^	12.22^a^	0.434	0.026

Values are means of two replicates and standard errors of means.

Within each variable, values with the same superscript letter are not significantly different according to Duncan’s multiple range test (P > 0.05).

NC−: unchallenged negative control; PC−: unchallenged positive control; Pro−: unchallenged probiotics control; Pro+: challenged probiotic control; PC+: challenged positive control; NC+: challenged negative control.

*HDL* high density lipid, *LDL* low density lipid, *RISK* total cholesterol-HDL ratio.

The results of the haematogical parameters analyzed are shown in Table 7. Whereas no difference (P > 0.05) was recorded in total RBC, haemoglobin, ESR, PCV, basophiles, monocytes, total platelet count and MPV across the treatments on day 21, statistical difference were recorded for total WBC and neutrophils between the probiotics supplemented groups when compared with other treatments. Nevertheless, on day 28 of the experiment MCV and RDW were significantly (P > 0.05) higher in probiotics supplemented groups Pro– and Pro+ when compared with non-*P. multocida* challenged negative control group, NC−. Also, probiotics supplemented birds with or without *P. multocida* challenge significantly (P < 0.05) increased total WBC counts at the end of the trial. Also, the concentrations of lymphocytes and monocytes were higher in probiotics supplemented groups while PC− and Prob− had higher (P > 0.05) total platelet counts on day 28 of the experiment.

**Table 7:** Effects of probiotics supplementation on haematological parameters of *Pasteurella multocida* challenged broiler chickens.

Parameter	Treatment							*P* value
	NC−	PC−	Pro−	Pro+	PC+	NC+	SEM	
**Day 21**								
RBC								
Total RBC (mil/Cmm)	1.68^a^	2.59^a^	2.71^a^	2.48^a^	2.62^a^	2.17^a^	0.158	0.310
Haemoglobin (g/dL)	6.65^a^	7.58^a^	7.45^a^	7.62^a^	7.3^a^	6.75^a^	0.318	0.092
ESR (mm/1 h)	3^a^	2^a^	3^a^	4^a^	2^a^	3.5^a^	0.327	0.420
PCV (%)	22.15^a^	33.4^a^	36.7^a^	34.3^a^	35.4^a^	29^a^	2.212	0.198
MCV (fl)	130.4^bc^	129.2^c^	142.1^ab^	144.25^a^	135.45^abc^	134.05^abc^	2.677	0.032
MCH (pg)	34.15^a^	30.2^bc^	32.4^ab^	33^ab^	28.05^c^	31.1^abc^	0.891	0.024
MCHC (g/dL)	26.25^a^	23.35^ab^	22.45^b^	22.9^a^	20.65^b^	23.2^ab^	0.741	0.040
RDW (%)	9.35^c^	9.35^c^	11.85^b^	10.15^c^	12.95^a^	9.75^c^	0.609	0.022
WBC								
Total WBC (C/mm)	63845^c^	171760^abc^	233860^ab^	266850^a^	123320^bc^	110985^bc^	19,785.52	0.001
Neutrophils (%)	40.5^a^	36.5^a^	34^ab^	32.00^ab^	23.5^b^	36^a^	5.567	0.013
Lymphocytes (%)	57^b^	59.5^b^	80^ab^	91.8^a^	73^ab^	58.4^b^	6.167	0.036
Monocytes (%)	1^a^	0.5^a^	2^a^	2.5^a^	1^a^	1^a^	0.307	0.415
Basophiles (%)	0.5^a^	0.5^a^	0.67^a^	1.27^a^	0.75^a^	0.78^a^	0.196	0.172
Platelets								
Total platelet count (C/mm)	3500^a^	2500^a^	2350^a^	2200^a^	2000^a^	2500^a^	202.76	0.113
MPV (fl)	8.9^a^	7.9^a^	10.8^a^	11.45^a^	11.65^a^	10.55^a^	0.609	0.081
**Day 28**								
RBC								
Total RBC (mil/Cmm)	2.08^a^	3.07^a^	3.08^a^	2.78^a^	2.615^a^	2.66^a^	0.150	0.107
Haemoglobin (g/dL)	6.51^ab^	6.65^ab^	8.78^a^	7.77^ab^	6.58^ab^	6.22^b^	0.230	0.035
ESR (mm/1 h)	3^a^	2^a^	3^a^	4^a^	2^a^	3.5^a^	0.327	0.195
PCV (%)	28.15^c^	30.4^bc^	36.7^c^	34.05^ab^	31.25^abc^	25.63^c^	1.383	0.004
MCV (fl)	129.8^c^	135.15^bc^	148.20^a^	141.80^ab^	135.59^bc^	126.9^c^	3.312	0.026
MCH (pg)	32.75^a^	29.3^a^	31.26^a^	33.2^a^	27.04^a^	30.16^a^	0.938	0.387
MCHC (g/dL)	23.45^a^	23.19^a^	22.75^a^	24.1^a^	24^a^	23.26^a^	0.210	0.410
RDW (%)	8.97^b^	10.61^ab^	10.98^ab^	11.38^a^	11.70^a^	9.54^ab^	0.437	0.031
WBC								
Total WBC (C/mm)	172070^c^	202866^c^	302440^ab^	346652^a^	235780^bc^	223933^bc^	26,314.05	0.001
Neutrophils (%)	39^a^	39.5^a^	35^a^	37^a^	31.5^a^	38.5^a^	1.243	0.201
Lymphocytes (%)	52.5^b^	57.7^b^	88.6^a^	93.5^a^	67^ab^	55.75^b^	7.566	0.021
Monocytes (%)	0.75^b^	1^b^	2.5^a^	3.5^a^	1.25^b^	0.75^b^	0.338	0.023
Basophiles (%)	1^a^	0.5^a^	1.25^a^	1.02^a^	1^a^	0.75^a^	0.106	0.719
Platelets								
Total platelet count (C/mm)	4000^ab^	5500^ab^	7500^a^	5250^ab^	3750^b^	2750^b^	681.35	0.019
MPV (fl)	11.25^a^	9.1^a^	9.95^a^	10.85^a^	11.15^a^	11.95^a^	0.417	0.231

Values are means of two replicates and standard errors of means.

Within each variable, values with the same superscript letter are not significantly different according to Duncan’s multiple range test (P > 0.05).

NC−: unchallenged negative control; PC−: unchallenged positive control; Pro−: unchallenged probiotics control; Pro+: challenged probiotic control; PC+: challenged positive control; NC+: challenged negative control.

*MPV* mean platelets volume, *MVC* mean corpuscular volume, *MCH* mean corpuscular haemoglobin, *MCHC* mean corpuscular haemoglobin concentration, *RDW* RBC distribution width, *ESR* erythrocyte sedimentation rate.

### Anti-inflammatory gene expression

On 14-day post *P. multocida* challenged, dietary supplementation of birds with probiotics significantly (P < 0.05) upregulated the mRNA profiles of anti-inflammatory genes including HIF1A (hypoxia inducible factor 1 alpha) and TSG-6 (Tumor necrosis factor- (TNF) stimulated gene-6) on the caecal mucosa when compared to the birds in the control group (Table 8). However, when both anti-inflammatory genes are compared, probiotic effect in the upregulating the expression of HIF1A was higher than for PTGER2. There was no difference in the expression of both anti-inflammatory genes in birds supplemented with antibiotic and the negative control except for TSG-6 (Table 8).

**Table 8:** Effect of probiotics supplementation on anti-inflammatory gene expression in caecal mucosa of *Pasteurella multocida* challenged broilers.

Anti-inflammatory gene	*Pasteurella multocida* infection				*P* value
	Treatment				
	Prob+	PC+	NC+	SEM	
**14 days post challenged (2**^**−ΔΔCt**^**)**					
PTGER2	1.13^a^	0.75^a^	1.02^a^	0.113	0.884
HIF1A	134.58^a^	36.82^b^	13.23^b^	37.148	0.011
TSG-6	13.71^a^	9.86^b^	1.05^c^	3.747	0.003

Values are means of two replicates and standard errors of means.

Within each variable, values with the same superscript letter are not significantly different according to Duncan’s multiple range test (P > 0.05). Pro+: challenged probiotic control; PC+: challenged positive control; NC+: challenged negative control.

*HIF1A* hypoxia inducible factor 1 alpha, *PTGER2* prostaglandin E receptor 2, *TSG-6* tumor necrosis factor- (TNF) stimulated gene-6, *Pro+* challenged probiotic, *PC+* challenged antibiotic; *NC+* challenged control.

## Discussion

Avian cholera caused by *P. multocida* is a highly contagious poultry disease of global concern, causing significant ecological and economic challenges to the poultry industry each year^3,24,25^. The ability of *P. multocida* to survive asymptomatically in carrier birds for a longer period of time even after the disappearance of clinical signs have often led to frequent recurrence of *P. multocida* outbreaks with high mortality^4,25^. Also, *P. multocida* has been reported to persist for several months in the environment, water supplies, and insects^24,26,27^. In this study, we proposed that the supplementation of poultry with multistrain probiotics containing *L. plantarum*, *L. fermentum*, *P. acidilactici*, *E. faecium* and *S. cerevisiae* can control *P. multocida* infection in broiler chickens through mitigating the manifestation of clinical signs and the reduction of mortality associated with *P. multocida* while improving the overall performance.

Although some studies have previously tried the effectiveness of vaccines as antibiotic alternatives in the control of *P. multocida* in poultry, studies evaluating the possible role of probiotics in the control of *P. multocida* colonization and infections in poultry production are lacking. With the successful reports of probiotics effectiveness in the control and mitigation of the colonization and infection by poultry pathogens including *Salmonella*^14,28,29^, Campylobacter^15,30,31^, *E. coli*^32,33^, *Eimeria* spp.^34,35^, *L. monocytogenes*^36–38^ and *Clostridium perfringens*^39,40^, the trial of probiotics in the control of *P. multocida* would further unravel probiotics effectiveness against this devastating poultry pathogen.

Generally, the dietary supplementation of probiotics has been reported to positively influence animal health and productivity. The results obtained from this study shows that dietary inclusion of probiotics significantly improved the performance and feed efficiency in broiler chickens with beneficial impact on the intestinal microbiota composition and health, hence decreasing the severity of the FC in birds. The significant improvement in BW, BWG and FCR recorded among broilers supplemented with probiotics in comparison with the controls from this study confirmed the positive impact of probiotics supplementation on the performance of broilers. This finding is significant not only to confirm the improvement of intestinal health after probiotic supplementation, but also to mitigate the economic losses due to *P. multocida* infections in poultry production. Our results agreed with the findings of Smialek et al., Massacci et al., Mountzouris et al., Olnood et al. who reported significantly improved growth performance and feed efficiency of probiotics supplemented broilers challenged with either *Campylobacter* and *Salmonella* respectively^14,15,28,30^. Furthermore, apart from the adoption of biosecurity measures and vaccination (which are most potent preventive measures) in endemic regions of the world, the routine use of probiotics in the control and prevention of enteric infections in poultry production could further help in reducing the severity of cases of fowl cholera hence, diminishing the spread of the pathogen.

Changes in the relative weight of visceral organs and carcass in broilers is one principal effect mostly attributed to probiotic supplementation in poultry. The symptoms of pasteurellosis in the *P. multocida* challenged positive control (PC+) and negative control (NC+) were accompanied by decrease in the live BW of birds, visceral organs and dressing carcass in these treatments with higher increase in gizzard weights on day 28 of the experiment. These trends were similarly reported previously by Olnood et al., and Park and Kim, after experimentally infecting broiler chickens with *Salmonella* spp.^41,42^. The improved growth performance of birds due to probiotic supplementation as observed in the current study was also reported by other researchers after challenging broilers with different pathogens. The dietary supplementation of broilers with *B. subtilis* as probiotic significantly increased the relative weight of spleen by 3.8% without significantly affecting the relative weights of liver and bursa of Fabricius^43^. In agreement with our study, Pedroso et al. reported that the dietary inclusion of *Lactobacillus reuteri* and *L. johnsonii* as probiotics significantly increased intestinal weight in 21-day old broilers^44^. Probiotics effect on the weight of visceral organs and intestines of animals is inexplicit, and can also be determined by the nature and amount of microbial strains used as probiotics. It has been reported that probiotics consistently influence the intestinal morphology and micro-structure which often increases the absorptive function of the ileum^14,45^. Also, Pelicano et al. reported significant improvement in the leg yield and breast of birds fed with probiotics^46^.

A significantly higher mortality was recorded in *P. multocida* challenged birds supplemented with antibiotic and challenged negative control when compared with the *P. multocida* challenged birds supplemented with probiotic. The change in behavior and the manifestation of clinical signs in the control groups were consistent with the reported signs characterizing fowl cholera in poultry such as diarrhoea, depression, listlessness, severe weakness, nasal discharge, recumbency and moribund status, isolation, anorexia combined with reduction in feed and water consumption, ruffled feathers, immobility and lameness^4,22,23^. Within 24 h post-*P. multocida* infection, birds in the control groups showed mild to moderate signs with mortality which increased in severity till about 92 h post infection. A similar trend was reported in experimental birds challenged with *P. multocida*^25,47,48^. Depending on the strain, the incubation period of *P. multocida* usually varied between 12 and 48 h with 100% mortality majorly between 24 and 72 h post infection^49^. There is generally limited information about the clinical pathology of pasteurellosis in poultry. Also, Wilkie et al. reported that broiler chickens experimentally challenged with *P. multocida* died within 22–72 h post infection^2^. Furthermore, the rapid death of the host animal including broilers due to acute form of fowl cholera is a characteristic of septicaemia induced by *P. multocida*^47^. The persistence of *P. multocida* strains at the site of infection as well as their migration to other host tissues and organs, and the eventual time of the host death depend primarily on the host immune response and the characteristics of *P. multocida* strain causing the infection which may also influence the shedding and isolation of the pathogen^2,47,50^. The inhibitory effect of each of the probiotic strain i.e. *L. plantarum*, *L. fermentum*, *P. acidilactici*, *E. faecium* and *S. cerevisiae* (used in this study) against *P. multocida* and other poultry pathogens have been evaluated previously and their probiotic potentials elucidated^17,18^.

Information regarding in vivo antimicrobial activity of probiotics strains including LAB and *Saccharomyces* against *P. multocida* and *P. multocida* infections are lacking. However, the inhibitory activity of probiotics consisting of strains of LAB and *S. cerevisiae* in broilers as recorded in this study indicates that these probiotic strains can be successfully used as alternatives for growth-promoting antibiotics in poultry production. The numbers of enterobacteria in the *P. multocida* challenged control groups were higher in the ileum and ceacum than the unchallenged groups both on days 21 and 28 of the experiment. Probiotic supplemented groups showed higher number of LAB and yeast in the gizzard, ileum and caecum which significantly decreased the numbers of *P. multocida* on both sampling days during the experiment. The reduction of enterobacteria by beneficial gut microflora may be attributed to the bacteriostatic effect of volatile fatty acids (VFA) secreted in the GIT of birds^14^. In vitro evaluation has demonstrated that VFA inhibited enterobacterial growth at the pH of 6^51^. Therefore, probiotics supplementation may have increased the concentration of VFA in the gut of the birds examined. This finding is in agreement with the results of Lan et al.^52^ who reported significant decrease in the number of enterobacteria after broiler chickens were supplemented with multi-strain probiotics containing a mixture of *L. agilis*, *L. acidophilus*/*gallinarum* and *L. salivarius*. The inclusion of strains of *Bacillus*, *Clostridium* and *Lactobacillus* as multi-strain probiotics at the level of 10^6^ to 10^9^ CFU/kg of diet reportedly suppressed the growth of enterobacteria^53–55^. Also, probiotics’ antimicrobial effects come from their secretion of antimicrobial compounds including bacteriocins, organic acids (acetic, lactic, propionic, succinic acid, etc.), short-chain fatty acids, hydrogen peroxide and other low molecular weight substances^56^. The combination of the strains of LAB and yeast used as multi-strain probiotic in this study possibly synergized to form a robust antimicrobial activity against *P. multocida* and enterobacteria in the gut of the birds supplemented with the study probiotics.

Furthermore, these probiotic strains are able to competitively exclude pathogens, hence preventing their attachment to intestinal walls thereby improving intestinal microbial balance. In agreement with Olnood et al. there was a gradual increase in pH concentration from the proximal to the distal GIT regions with probiotic supplemented birds having a more lowered pH level especially in the gizzards^14,41^. The reduced pH among probiotic supplemented broiler chickens also contributed in the reduction of the numbers of *P. multocida* and enterobacteria.

Dietary conditions and pathological stress commonly determine the haematological changes and health status of birds^57^. The reduction in major haematological parameters including Hb, PCV, ESR and total RBC in *P. multocida* challenged control groups clearly depicts the onset of anaemia. The occurrence of anaemia in avian cholera infection in poultry has been properly reported^48^. The cause of anaemia in *P. multocida* challenged birds as recorded in this study may be attributed to bacterial septicaemia. The concentration of total WBC and lymphocytes were also higher in probiotics supplemented birds. Probiotics are known to modulate host immune system response primarily through balance between anti-inflammatory and proinflammatory cytokines^36^. Similarly, after dietary supplementation of *B. subtilis*-based probiotics, Park and Kim and Lee et al. showed the reduction of coccidiosis clinical signs and improved immune response in broiler chickens challenged with *Eimeria maxima*^42,58^. The improvement of gut health through the modulation of gut microflora and the modulation of intestinal inflammatory and immune response may significantly inhibit the *P. multocida* colonization and proliferation within the gut hence influencing haptoglobin concentration. The dietary inclusion of probiotics positively influenced haematopoiesis which among others increase the WBC counts, hence enhancing immune cells synthesis which further protects the host against invading pathogens^59,60^. The presence of congested blood vessels and haemorrhages observed in the lungs, livers, hearts and intestines of *P. multocida* infected birds as a result of fowl cholera is similar to the findings of Shivachandra et al. and Sonone et al.^9,48^. The supplementation of probiotics as revealed in this study significantly reduced the severity of *P. multocida* infection throughout the experiment, hence, reflecting in the improved haematological parameters as clearly shown.

The reduction in the concentration of total cholesterol, triglycerides, glucose and LDL cholesterol which are major biochemical parameters as reported in our study due to probiotic supplementation agrees with the report of Arun et al. and Al-Kassie et al. who separately reported a significant reduction in total cholesterol, triglycerides and glucose by dietary inclusion of 100 mg/kg diet of *L. sporogene* probiotic and the combination of probiotic (*Aspergillus niger*) and prebiotic (*Taraxacum officinale*) in broilers^61,62^. Total cholesterol reduction in probiotic supplemented birds could be as a result of direct assimilation of cholesterol by bacterial cells (which causes reduction in the cholesterol absorption and synthesis in the GIT), 3-hydroxy-3-methyl-glutaryl-CoA reductase inhibition and bile salt hydrolysis^63,64^. Furthermore, triglyceride reduction in probiotic treated birds may be as a result of increased hydrolysis of bile salt which causes inadequate lipid absorption in the small intestine^10^. Strains of *Lactobacillus* are known to show high hydrolytic activity on bile salt which consequently leads to bile salts deconjugation within the GIT^65^. Also, the concentration of total protein was significantly higher in probiotics fed birds. This corroborated with the findings of Dimcho et al. and Alkhalf et al. who reported probiotic effects on total protein concentration in chickens^10,66^.

Maintaining intestinal health and the integrity of intestinal barrier function is essential for the growth and wellbeing of animals. Several pathogenic factors such as stress and pathogenic bacteria challenges can cause inflammation and damage to the intestinal barrier^67^. On day 14 post *P. multocida* challenge, greater effects were found for probiotic supplementation on HIF1A, and TSG-6 (P < 0.05). The data obtained from this study implies that probiotics supplementation can attenuate inflammatory reactions through the upregulation of the secretion of anti-inflammatory factors.

In the gut of animals, pathogens including *P. multocida* readily cause different degree of inflammatory damage of intestinal epithelial cells by establishing hypoxic microenvironments^68^. One of the major transcriptional factors that dampen hypoxia-induced inflammation in the gut is HIF1A. HIF1A enhances the synthesis and signaling effects of anti-inflammatory signaling molecules^69^. TSG-6 is multifunctional protein that has been implicated as having important anti-inflammatory and tissue protective properties^70^. From this study, HIF1A and TSG-6 in the control treatment with *P. multocida* infection expressed the lowest mRNA profiles, whereas they were upregulated with probiotics supplementation, depicting that these 2 genes are collaboratively associated with either *P. multocida* or probiotics. This is the first work that assessed the expression of these 2 genes on probiotics supplemented chickens infected with *P. multocida*. Unfortunately, no information exists about the expression of these genes in the presence of probiotics and *P. multocida*. However, in a recent work Deng et al. reported the upregulation of HIF1A in probiotic supplemented chickens infected with *Listeria monocytogenes*^36^. Therefore, further study on the mechanisms responsible for the dampening of inflammation and the upregulation of anti-inflammatory factors, HIF1A and TSG-6 in probiotics supplemented birds is needed. The higher population of Enterobacteria and *P. multocida* in the control group could be associated with the invasion of these bacteria and the pathogenesis of *P. multocida* resulting in the clinical manifestation of *P. multocida* infection and high mortality. A pool of *P. multocida* strains could be evaluated to determine their persistence, spread and multiplication in host tissues and shedding in future research.

## Materials and methods

### Ethical approval of the study

The field trial was approved by the Animal Care and Use Committee of the Faculty of Biological Science and Technology, Jashore University of Science and Technology, Jashore, Bangladesh (certification number: ERC/FBST/JUST/2019-32). The health status of birds in the field were routinely monitored by a veterinarian. The birds were kept under controlled environmental conditions in the animal house of Jashore University of Science and Technology, Jashore, Bangladesh, throughout the experimental period. Authors confirmed that all experiments were performed in accordance with relevant guidelines and regulations and in compliance with the Animal Research: Reporting of in vivo Experiments (ARRIVE) guidelines.

### Strains used for the study and diets

Five potential probiotic strains previously isolated from broiler chicken and raw milk, and identified using 16S rRNA sequencing as *Lactobacillus plantarum*, *L. fermentum*, *Pediococcus acidilactici*, *Enterococcus faecium* and *Saccharomyces cerevisiae* with suitable probiotic properties including antagonistic activity against broad range of poultry pathogens including *P. multocida*, survivability in simulated gastric juice, bile salts and phenol tolerance, adhesion to ileum epithelial cells, aggregation and hydrophobicity abilities, α-glucosidase inhibitory activity, and competitive exclusion of pathogens were selected for this field trial^17,18^. Basal diets (starter and grower/finisher) formulated in our laboratory were provided as pellets all through the trial and were based on wheat, soybean meal and corn as shown in Table 9. The five probiotic strains were mixed at equal ratio and added into respective experimental treatments at dose of 10^8^ CFU/kg of diet.

**Table 9:** Ingredient composition and calculated nutrient of basal diet used for the study.

Item	Starter	Finisher
**Ingredient, g/kg**		
Wheat (26.00%)	262.00	214.00
Sorghum (15.5%)	350.25	400.00
Mung beans	100.00	100.00
Soybean meal (46%)	157.00	81.50
Limestone	15.50	16.00
Salt	1.75	1.50
Soybean oil	2.00	2.50
Moisture	8.00	6.70
Ash	7.60	6.00
Dicalcium phosphate	1.50	1.50
Lysine	2.10	0.40
Methionine	2.10	1.30
Threonine	0.20	0.15
Vitamin and mineral premix*	2.50	2.50
Crude protein	200.00	190.00
Crude fibre	35.17	43.14
Crude fat	52.16	54.47

*Nutrition value per Kg of vitamin and mineral premix contains 140,000 IU of vitamin A, 70 mg of vitamin E, 3000 IU of vitamin D3, 4 mg of vitamin K, 3 mg of thiamine, 10 mg of vitaminB2, 8 mg of vitamin B6, 0.04 mg of vitamin B12, 48 mg of niacin, 20 mg of calcium d-pantothenate, 500 mg of choline chloride, 0.20 mg of biotin, 1.8 mg of folic acid, 80 mg of manganese, 70 mg of zinc, 50 mg of iron, 10 mg of copper, 3 mg of iodine, 0.4 mg of selenium, and 0.2 mg of cobalt.

### Experimental design and treatments

**Figure 2:**
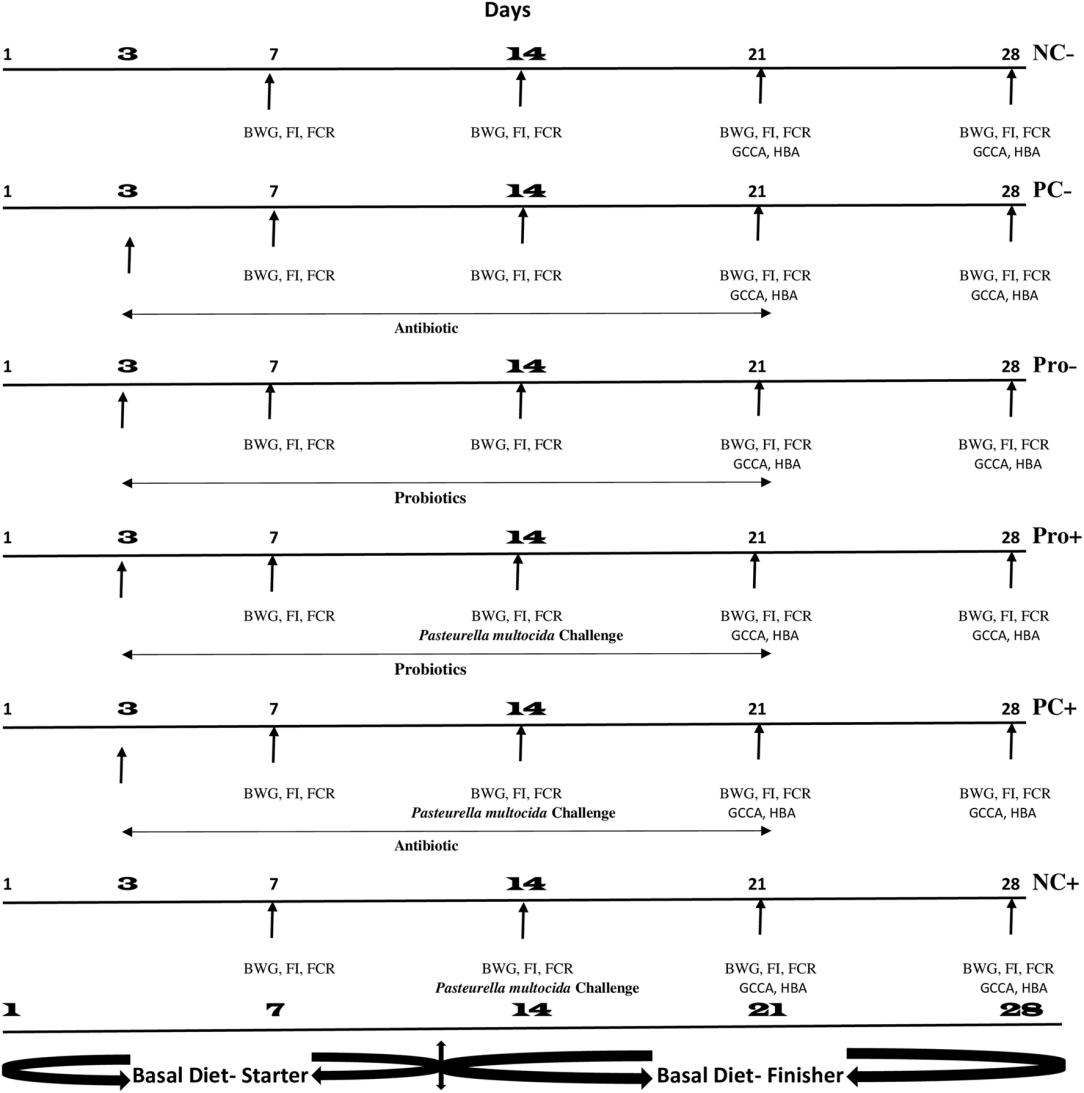
Schematic illustration of the experimental design. Birds were fed basal diet all through the experiment. Treatment groups included: NC−: unchallenged negative control; PC−: unchallenged positive control; Pro−: unchallenged probiotics control; Pro+: challenged probiotic control; PC+: challenged positive control; NC+: challenged negative control. *BWG* body weight gain, *FCR* feed conversion ratio, *FI* feed intake, *GCCA* gut content and carcass analysis, *HBA* haemato-biochemical analysis.

A total of 120 one-day-old Cobb 500 broiler mixed-sex chicks were purchased from a commercial hatchery (NOURISH FARMS, DHAKA, BANGLADESH), weighed individually and randomly assigned to 6 experimental treatments with 2 replicate groups containing 10 chicks each after they were allowed to acclimatize for 2 days. Birds in each treatment were housed in a floor pen containing sawdust litter. Twenty-three hours of light was provided during the first week and then reduced to 18 h throughout the 28 days of the experiment. The 6 experimental treatments adopted in this trial included: (1) negative control (NC−), non-probiotic and unchallenged with *P. multocida*; (2) positive control (PC−), supplemented with doxycycline HCL (0.5 g/mL), non-probiotic and unchallenged with *P. multocida*; (3) probiotic control (Pro−), probiotics supplemented and unchallenged with *P. multocida*; (4) probiotic challenged (Pro+), as probiotics supplemented and challenged with *P. multocida*; (5) positive challenged (PC+), as doxycycline HCl, supplemented and *P. multocida* challenged; and (6) negative challenge (NC+), as non-antibiotic, non-probiotic and challenged with *P. multocida*. In all the treatments, feed and water were provided ad libitum according to the experimental design. Birds in the antibiotic treatments were administered 1 g/L of the doxycycline HCL following the manufacturer’s instructions. Both probiotics and antibiotic were administered between days 3–21 of the experiment (Fig. 2).

### Pathogen challenge

For the pathogen challenge experiment, strain P-931of *P. multocida* subsp. *Multocida* ATCC 12945 (capsular Type A) isolated from fowl and known to cause fowl cholera was obtained and used for the experimental infection of birds. Stock culture of *P. multocida* stored at − 80 °C was revived and grown overnight at 37 °C in brain heart infusion broth (LIOFICHEM, ABRUZZI, ITALY) with shaking at 150 rpm. Cells were harvested by washing (8000×*g*, 10 min) three times with sterile PBS and finally reconstituted in PBS to 10^8^ CFU/mL^4^. At day 14 of the trial, each bird in experimental challenged treatments was orally inoculated with 1 mL of 10^8^ CFU of *P. multocida* as previously described^2^. Birds were examined at 12 h intervals for typical clinical manifestations of fowl cholera until the end of the experiment. Clinical signs and mortality were recorded accordingly. Major clinical signs and symptoms characterizing fowl cholera that were examined included nasal discharge, diarrhoea, lameness, weakness, moribund state, reduced activity, reduced water and feed intake, ruffled feathers and rapid breathing^4,49^.

### Sample collection and processing

The individual weight of all the chickens were measured before grouping them into respective treatment pens. Individual bird and leftover feed from each treatment were weighed weekly and the feed intake (FI) and body weight gain (BWG) recorded. Also, feed conversion ratio (FCR; feed intake/weight gain) and mortality (when it occurred) for each treatment were also calculated^41,71^.

On days 21 and 28 of the experiment, four birds from each pen were selected at random and sacrificed by cervical dislocation after exposing them to overdose of isoflurane anesthesia. All efforts were made to minimize suffering. Visceral organs of each of the sacrificed bird were carefully removed and weighed after opening the abdominal cavity. After emptying the contents into sterile plastic containers, the weight of gizzard, ileum and caecum were recorded. Also, the weight of heart, liver, bursa, spleen, thigh, drumstick, breast, wing and dressing were recorded and expressed as the percentage of the body weight^43^.

### Enumeration of intestinal bacteria, yeast and detection of *P. multocida*

For each bird sacrificed, fresh gizzard, ileum and caecum digesta were immediately collected within 1 h for microbial enumeration. Using 0.85% normal saline solution, approximately 1 g of the fresh digesta samples were serially diluted for the enumeration of total aerobes, enterobacteria (coliforms and lactose negative enterobacteria), lactic acid bacteria, total yeast and *P. multocida* by conventional microbiological techniques using selective media including nutrient agar (LIOFILCHEM, ITALY), MacConkey agar (LIOFILCHEM, ITALY), De Man, Rogosa and Sharpe (MRS) agar (LIOFILCHEM, ITALY), Sabouraud dextrose agar (LIOFILCHEM, ITALY) and 5% defibrinated sheep blood agar supplemented with amikacin (2 mg/L), vancomycin (4 mg/L), and amphotericin B (4 mg/L) respectively^72,73^. Microbiota enumerations were conducted in duplicate and the average determined. Results of the bacterial counts obtained were expressed as log_10_CFU/g (base-10 logarithm colony-forming units per gram) of gizzard, ileal and caecal digesta.

### Determination of digesta pH

Exactly 1 g of fresh digesta samples from gizzard, ileum and caecum of each sacrificed bird on days 21 and 28 were transferred into 9 mL of distilled water in 15 mL tubes and vigorously mixed. After thorough stomaching, the suspension was mixed with a stirrer after which the pH was measured using glass electrode (HANNA INSTRUMENTS, INC., WOONSOCKET, RI, USA)^74^.

### Haemato-biochemical parameters

Complete blood counts and lipid profile determining the haemato-biochemical parameters were carried out. Approximately 4 mL of blood samples from each bird sacrificed were collected from the jugular vein into plane tubes (for biochemical analyses) and anticoagulant tubes (for haematological analysis) on days 21 and 28 of the trial. Haematological assays were conducted using automatic SYSAM-XN-1000, XN-550 AL Random Access Haematology Machine (SYSMEX CORPORATION, JAPAN) and checked manually while the biochemical analyses were carried out by Siemens Dimension RxL/Max/Vitros350 Random Access Chemistry Analyzer (SIEMENS HEALTHCARE DIAGNOSTICS INC, USA) after obtaining the serum through centrifugation. The average of results obtained from the haemato-biochemical analyses per treatment were determined.

### Total RNA extraction and mRNA quantification

Approximately 1 g of caecal mucosa was aseptically collected from each of the sacrificed bird for gene quantification. Total RNA was isolated from the caecal tissues samples using the DIRECT-ZOL RNA extraction Kits (ZYMO RESEARCH, IRVINE, USA), following the manufacturer’s protocol, and finally using a Nanodrop 2000 spectrophotometer (THERMO SCIENTIFIC, WILMINGTON, DE), the concentration and purity of RNA were quantified. The purified RNA was converted into complementary DNA (cDNA) using ProtoScript II First Strand cDNA Synthesis Kit (NEW ENGLAND BIOLABS, INC., MASSACHUSETTS, USA) following procedure described by the manufacturer. Real-time qPCR was performed with the BIO-RAD CFX96 Real-time PCR system (BIO-RAD LABORATORIES, CA, USA). The target genes and primers sequences^36^ are shown in Table 10. The PCR reaction was conducted using POWERUP SYBR Green Master Mix (THERMO FISHER SCIENTIFIC INC., MASSACHUSETTS, USA) consisting a total of 20 μL PCR reaction-mix containing 10 μL 2× SYBR Green PCR Master Mix, 2 μL primers (0.5 μM of 1 μL forward and 1 μL reverse primer) 6 μL template cDNA (~ 10 ng/μL) and 2 μL nuclease-free water. The qPCR cycling condition consisting of initial heat activation at 95 °C for 10 min, following by 40 cycles of denaturation at 95 °C for 15 s, annealing at 58 °C for 30 s and finally 30 s of extension at 72 °C. The optimum annealing temperature of target and reference genes were determined by the gradient protocol of BIO-RAD CFX96 Real-time PCR System (BIO-RAD LABORATORIES, CA, USA). The procedure by Livak and Schmittgen^75^ was used to determine the transcriptional profiles of specific gene of interest and were expressed as the relative expression to the housekeeping gene used (2^−ΔΔCt^).

**Table 10:** Primer sequences used for RT-qPCR.

Gene	GenBank	Sequence 5′ → 3′	Length (bp)
HIF1A	NM_204297.1	F-CCAGCAGTTCCTCATGCAAT	215
		R-AAATGCTGCTAGCCCTTCCC	
PTGER2	NM_001083365.1	F-TTGCACGTCACCTTCTCGTT	235
		R-TGATGGTCATGATGGCGAGG	
TSG-6	DQ275160.1	F-ATGGACAGCGGATTCACCTC	219
		R-TCTGAAACCCACCAGCAGTC	
ACTB	NM_205518.1	F-TTACTCGCCTCTGTGAAGGC	228
		R-TCCTAGACTGTGGGGGACTG	

*ACTB* beta-actin, *HIF1A* hypoxia inducible factor 1 alpha, *PTGER2* prostaglandin E receptor 2, *TSG-6* tumor necrosis factor- (TNF) stimulated gene-6, *F* forward, *R* reverse.

### Statistical analysis

Data were collected and analyzed by analysis of variance as a completely randomized design using the GLM procedure as described by GRAPHPAD PRISM version 5.0 for Windows (GRAPHPAD SOFTWARE, SAN DIEGO, CA, USA) and SAS software (version 9.4, SAS Institute Inc., Cary, NC). Viable counts of the gizzard, ileum and caecum contents were subjected to logarithmic conversion (Log_10_) before statistical analysis. All the results were presented as means of two independent experiments, and differences between treatment groups were determined using the Duncan’s multiple range test. Probability values less than 0.05 (P < 0.05) was considered as significant.
